# Value-related attitudes towards mental health problems and help-seeking barriers: a sequential mixed-methods design investigating participants with reported depressive episodes in rural Northern Germany with and without treatment experience

**DOI:** 10.1186/s12888-024-05521-9

**Published:** 2024-02-22

**Authors:** Karsten Valerius, Linnéa von Eitzen, Mirjam Göbel, Heike Ohlbrecht, Neeltje van den Berg, Henry Völzke, Hans J. Grabe, Georg Schomerus, Sven Speerforck

**Affiliations:** 1 Department of Psychiatry and Psychotherapy, Medical Faculty, Leipzig University, Leipzig, Germany; 2 Department of Education and Psychology, Freie Universität Berlin, Berlin, Germany; 3 Department for Social Sciences, Otto-von-Guericke University, Magdeburg, Germany; 4 Institute for Community Medicine, University Medicine Greifswald, Greifswald, Germany; 5 Department of Psychiatry and Psychotherapy, University Medicine Greifswald, Greifswald, Germany

**Keywords:** Help-seeking, Barriers, Mental health, Depression, Rural, Social milieu, Values, Masculinity, Qualitative, Grounded theory

## Abstract

**Background:**

Seeking help for severe depressive symptoms remains a major obstacle for particular groups within the general population. Value-related attitudes might contribute to this treatment gap, particularly in rural regions with a low density of psychiatric-psychotherapeutic services. We aimed to investigate narratives of socialization, value systems, and barriers of help-seeking to better understand social milieus at increased risk for underuse of psychiatric-psychotherapeutic services in a rural area in East Germany. This could complement the explanatory power of classical socio-demographic determinants and provide guidance for possible interventions.

**Method:**

Based on results of an analysis of a population-based German cohort study (SHIP-TREND-1), 20 individual semi-structured interviews were conducted with participants who met criteria for having been moderately or severely depressed at least once in their life. Qualitative analyses of interview data were guided by grounded theory methodology.

**Results:**

Participants with severe symptoms of depression were more frequent among non-responders of this study. We identified key aspects that influence help-seeking for mental health problems and seem to be characteristic for rural regions: family doctors serve as initial contact points for mental health problems and are considered as alternatives for mental health professionals; norms of traditional masculinity such as being more rational than emotional, needing to endure hardships, embodying strength, and being independent were frequently mentioned as inhibiting help-seeking by middle-aged men; anticipated adverse side-effects of therapy such as worsening of symptoms; a frequently expressed desire for less pathologically perceived treatment options.

**Conclusions:**

Our results suggest that barriers regarding help-seeking in rural regions are multifaceted and seem to be influenced by traditional norms of masculinity. We believe it is critical to strengthen existing and already utilized services such as family doctors and to implement and evaluate tailored interventions targeting the needs of the rural milieu.

**Supplementary Information:**

The online version contains supplementary material available at 10.1186/s12888-024-05521-9.

## Background

Against the backdrop of the high prevalence of mental illness in the German population [1], psychiatric care research aims to ensure the earliest and most adequate treatment possible for those with mental health problems. Despite a small, but significant decrease in non-help-seeking in a German representative population sample with prevalent mental health problems (from 62 to 57% between 1997 and 1999 and 2009–2012), more than half of those affected had not sought professional help in their lifetime at the time of the survey [2]. Such persistent reluctance to seek help is likely to have adverse impacts on mental health, such as worsening symptoms or slower recovery. Regarding depressive disorders, a review suggests that a maximum of one-third of people with suicidal ideation seek professional help [3]. Consequently, investigating barriers for those affected (e.g., stigmatization, structural obstacles, lack of suitable services, certain attitudes and values) is of particular relevance [4]. A qualitative synthesis of patients’ experiences investigating help-seeking behavior in depression concluded that several interrelated major concepts like help-seeking as a threat to identity, alternative coping strategies and multiple relational, structural, attitudinal, cognitive, culture-specific, or gender-specific barriers are of great importance [5]. Attitudinal barriers to care are particularly important in areas where help-seeking is already hindered by structural barriers such as a low density of psychiatric services. In this study we interviewed participants from Western Pomerania, a region with a socialist GDR^1^ background and a low density of psychiatric-psychotherapeutic services. Apart from two small towns (Greifswald and Stralsund, < 60 000 inhabitants), Western Pomerania can be considered a rural region with a low settlement density and a significantly lower density of professional psychiatric-psychotherapeutic services than many regions in the western part of Germany [6]. Since, according to projections, Western Pomerania’s current age structure already corresponds to the age structure of Germany in 10 to 20 years, to a certain extent, it can be understood as a potentially relevant model region for other structurally weaker regions in (East) Germany [7, 8].

Understanding the complex social processes influencing the use of psychiatric services against the background of a social milieu has been a scarcely used approach in psychiatric care research [9]. Consistent with the observation that qualitative data can provide a more nuanced and detailed picture of the multifaceted process of help-seeking [10], in-depth interviews were conducted. With a qualitative approach we want to complement the informative value of classical socio-demographic variables like age, gender and education. We explored narratives of socialization along with explicit and derived implicit values to gain a deeper understanding of the rural social milieu at greater risk of avoiding the use of mental health services. The term ‘socialization’ relates to the psychological developmental processes, through which individuals acquire and form their values, norms, ideologies, motivations and behaviors that they need to participate as capable members of society [11]. In light of this definition, the way individuals were socialized, overarching value orientations such as conformity to conservatism could shape attitudes towards help-seeking, i.e. value-related attitudes, and influence respective help-seeking behavior.

It remains unclear as to how social milieus with characteristic socialization and certain value systems differ regarding the acceptance of psychiatric services and in what ways the acceptance of psychiatric services could be increased through milieu-specific communication and support. Therefore, the goal of this study is to identify potential characteristics of a rural milieu that might help to develop future target-group specific approaches to reduce relevant barriers to treatment, especially regarding attitudes and knowledge.

## Methods

### Design, sample and recruiting

**Fig. 1:**
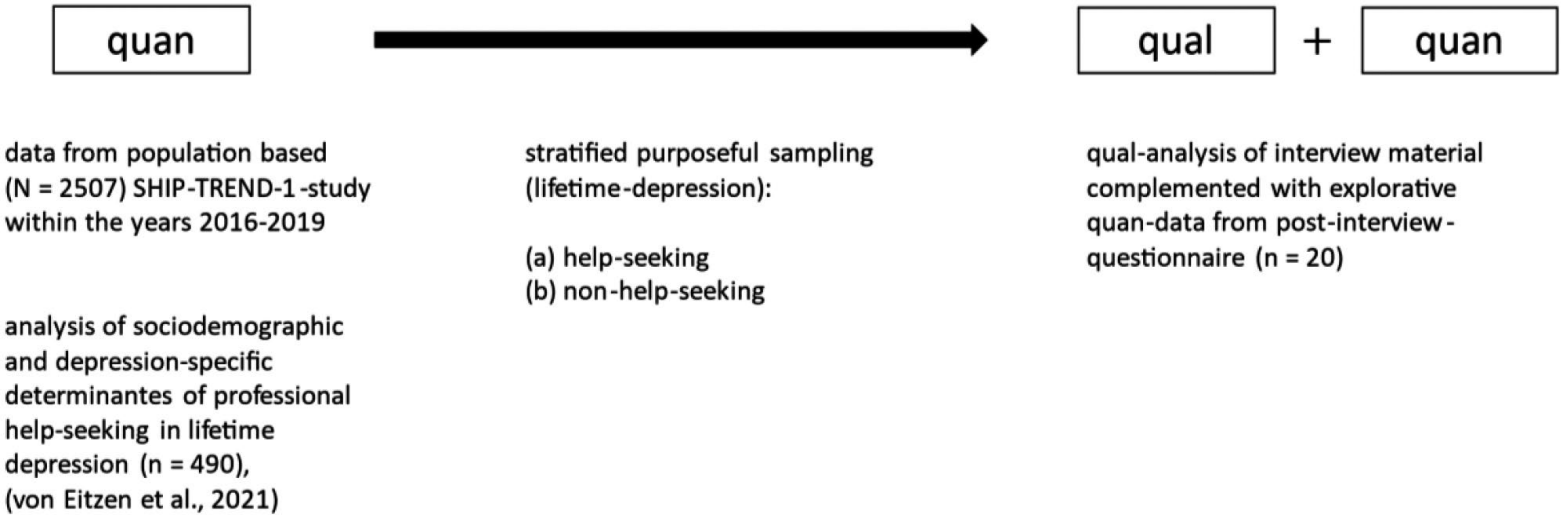
Exploratory sequential mixed-methods design (qualitatively driven). Figure 1 adopted from [14]

The present study was designed as a qualitatively driven sequential mixed-methods design [12, 13] with exploratory characteristics (see Fig. 1 for a schematic overview). Based on data of a population-based cohort study on the prevalence of diseases and their risk factors in Western Pomerania, (SHIP-TREND-1, data collection from 2016 to 2019), sociodemographic and depression-specific determinants of professional help-seeking in lifetime depression were investigated [7, 15]. Subsequently, 20 individual semi-structured face-to-face interviews (except for one interview that had to be done by phone) were conducted in a two-month period from August to September 2020. A post-interview questionnaire was designed to gain up-to-date information on help-seeking behavior since SHIP-TREND-1 and explore quantitative information about the interviewees after the interview (e.g., Schwartz’ Portrait Value Questionnaire (PVQ), Self-identification of mental illness scale (SELF-I), political orientation, conforming to masculinity). Due to the small number of participants these data were not analyzed using inferential statistics.

Stratified purposeful sampling was used to recruit the interviewees for the present study [7]. Potential interviewees for our study had already participated in the population-based cohort study SHIP-TREND-1 and met the criteria for having been moderately or severely depressed at least once in their life, i.e. lifetime depression. Of the 490 SHIP-TREND-1 participants who met the criteria for having been moderately or severely depressed at least once in their life, 54 potential interviewees were selected. For the purpose of our study, we oversampled participants who did not seek professional help (*n* = 40) compared to participants who sought professional help (*n* = 14).^2^ We balanced for gender, age and severity of depression (moderately or severely depressed) before potential interviewees were randomly selected.

Contrary to the grounded theory process of theoretical sampling, participants for the interview study were invited simultaneously in June 2020 via letter mail as part of a follow-up survey to the regional epidemiological SHIP-TREND-1 study. Due to data protection laws, recruitment of participants for the interviews was carried out by the SHIP-study centre and could not be done from interview to interview. One potential interviewee could not be contacted due to relocation, leaving 53 potential interviewees. In total, 20 participants that had reported a moderate or severe depressive episode in the past replied and participated in the interview study: 15 non-help-seeking participants and 5 help-seeking participants (see Table 1 for descriptive statistics). The study was approved by the ethics committee of the University of Greifswald (BB 038/18).

### Interview guideline

Latent aspects such as socialization, lifestyle orientation and values were explored in face-to-face interviews to assess barriers for professional help-seeking. Grounded theory methodology with explorative and interpretative strategies was chosen for the analysis of the collected interview data.

In light of our research questions, a semi-structured interview guideline was developed using a four-step approach [16]. The steps included (1) brainstorming and unrestricted collection of questions associated with the research topic, (2) reviewing the questions with regards to suitability, e.g., implicit expectations of researchers and openness, (3) sorting questions and key points by content and (4) subsuming the sorted bundles of questions and key points into narrative prompts.

The draft of the interview guideline underwent a reviewing process comprising five researchers: two psychologists and research associates employed in the project (KV, LvE), one Associate Professor and psychiatrist with a focus on psychodynamic-based psychotherapy (SSp), one psychologist not associated with the project, and a Professor of Sociology with experience in qualitative social research with a focus on health-related issues (HO). The reviewed interview guideline contained five main topics: (A) understanding of health and knowledge regarding mental health (e.g., “What do you generally understand by the term ‘health’?”), (B) relationship with medical practitioners and somatic treatment experience (e.g., “What have been difficult experiences for you in connection with medical treatments?”), (C) coping with problems, help-seeking and psychotherapy (e.g., “If you think back to the last situation you can remember in which you felt very sad or helpless: what was that like for you? What did you do?”), (D) social environment and mental health (e.g., “What are your experiences with mental health problems in your circle of acquaintances?”), (E) values and lifestyle orientation (e.g., “Judging from your own life experience, what advice would you give younger people for their life/their health?”). Each section was structured by guiding questions acting as a narrative prompt, maintenance questions helping to prolong the narrative flow as well as specific follow-up questions designed to shed light on aspects not mentioned that were of interest to the research.

After the first five interviews, the guideline was collaboratively adapted. Individual (follow-up) questions were optimized and questions regarding the formation of certain values through upbringing and socialization were added. Topic B was completely removed, since answers often resulted in excessive descriptions of somatic medical histories and undermined the intended purpose of easing discussion of help-seeking and psychotherapy-related questions. Thus, the final and adjusted interview guideline consisted of four main topics in respective sections which helped to adaptively conduct the interviews (see supplementary material A).

### Interviews

In order to practice formulating questions and placing narrative prompts, three trial interviews with one patient diagnosed with a major depression and two colleagues were carried out by KV (male) prior to conducting the interviews with study participants. Face-to-face interviews were originally meant to take place in the interviewee’s homes. This setting was planned to facilitate free and authentic statements regarding the interviewee’s relevance system and to allow for interviewer observations about the individual’s living environment. However, due to the COVID-19-pandemic, one interview was conducted via telephone and 19 interviews took place in a medical examination room at the SHIP study center of the Research Network Community Medicine Greifswald. Face-to-face interviews complied with strict hygiene regulations. KV conducted 18 of 20 interviews, including one telephone interview, and two interviews were conducted by LvE (female). The interviewers did not know in advance whether the interviewees had already sought help for mental health problems or not. Following informal light conversation and standardized introductory words (about interviewer’s background, overarching aims of the research and topics of conversation), oral clarification was given about the procedure and the rights of study participants. Subsequently, the interview guideline was followed. All interviews were recorded, with only the respective participants and interviewer present. During the interview, the interviewer took occasional notes on reported content and possible follow-up questions. The interview duration was around 95 min on average, with a range from 64 to 130 min. Directly following the interview, interviewees could take a break and then filled out a post-interview paper-pencil questionnaire, which lasted 10 to 15 min. All participants received 50€ as compensation for their efforts. The recordings of the 20 interviews were transcribed by MG, a sociologist, with the transcription software f4 (Version 7.0.6 Edu) according to extended transcription guidelines [17].

### Qualitative analysis based on grounded theory methodology

The study was guided by the principles of grounded theory methodology, with the research topic focusing on help-seeking. The epistemological basis of this research acknowledges different versions of grounded theory with respective assumptions and shared core principles [18, 19]. Through a critical realist perspective, the presented research is based on critical grounded theory, which presumes the existence of an objective reality that can only be described through filters of language, meaning-making, and social context. Critical grounded theory involves prior consideration of the issue at hand, contains inductive, deductive as well as abductive reasoning, and can be intended to bring about change to social structures [18, 21–22]. Grounded theory methodology is considered suitable for action and process based research questions and for developing middle-range theories [19, 23]. Middle-range applicability of exploratory findings was considered adequate for the future planning of a pilot intervention with the goal of improving communication about general existing mental health services and to develop support options specific to the target group.

Theoretical sampling according to grounded theory methodology could not be followed throughout the recruitment process as we had to comply with high data protection requirements and regulations of the SHIP-study center, which managed access to and group invitation of participants. Due to external restrictions, it is sometimes impossible to follow theoretical sampling. However, the idea of the iterative-cyclical process of theoretical sampling in grounded theory methodology can be approximately maintained by deciding which of the already transcribed interviews to analyze next based on initial analysis results [24]. We adopted Kruses advice on how to cope with external restrictions regarding theoretical sampling and jointly decided which interview transcript should be analyzed next. Our choice for which interview we were going to analyze next was guided by maximizing the contrast for observed phenomena that emerged in the prior interviews, e.g., coping styles for mental health problems, attitudes towards psychiatric help and motives for help-seeking.

The process of analyzing the data of this study was influenced by practical methods already suggested by Strauss and Corbin [23]. Transcripts were thoroughly read several times and initial codes were assigned using qualitative data analysis-software (MAXQDA Plus 2020), focusing on content broadly related to help-seeking and associated barriers, values, and socialization. The open coding phase was conducted equally by LvE and KV. The open coding step and the coding paradigm [23] allowed researchers to familiarize themselves with the data by gaining insights into action-related phenomena of help-seeking; see exemplary supplementary materials B and C (coding paradigms) and D (coding tree).

Further analyses of the transcripts and formation of relevant categories were jointly conducted by LvE (female), MG (female) and KV (male) in an iterative-cyclical process in which various phenomena and aspects that emerged from the transcripts were continuously compared. The comparison involved (a) revisiting transcripts, (b) reconsidering and revising initially formed categories, (c) interactively visualizing and comparing different concepts and reflections with the visual online collaboration platform MURAL, and (d) one or two weekly team meetings over a period of four months in which the concepts and their relevance against the backdrop of the underlying research questions were discussed. To support the dynamic process of constant comparison and data abstraction in a team of researchers, short summaries for each of the 20 transcripts and presumably important phenomena that emerged were prepared and continuously adjusted. In regularly scheduled meetings with SSP (male), who acted as an advisor and reviewer to the process of analyzing interview material, we presented evolving findings and matched the concepts to already existing theoretical constructs, thus leaving the path of solely inductive grounded theory. In addition to the project team, preliminary results were shared and discussed with experts in qualitative research methods from the Department of Microsociology at Otto von Guericke University Magdeburg. After five months of intensive analyses and multiple internal and external feedback loops, we did not discover any new relevant phenomena in the interview data. As a result, we held a final one-day evaluation workshop (with KV, LvE, MG and SSp) were we jointly determined which phenomena were most relevant to elaborate and report in further detail.

To report the methodology and results of our qualitative study we used the COREQ (Consolidated Criteria For Reporting Qualitative Research)-checklist (28 of 32). The full checklist can be found in the supplementary material E.

## Results

### Descriptive variables

The response of the originally invited sample (*n* = 53) was 37%. The mean age of the responder sample (*n* = 20), i.e. the interviewees, was 55.3 years (SD = 13.08, range from 29 to 86 years). This is around eight years older than the mean age in the state of Mecklenburg-Western Pomerania of 47.4 years [25]. People who responded to our invitation were more often male (65% of the responder sample). We also found explorative clues that responders were more likely to report lifetime depression with less severe symptoms than non-responders. Compared to responders, approximately twice as many non-responders fulfilled criteria for a severe depressive episode in their lifetime. Similarly, responders also reported a lower mean score on PHQ-9 than non-responders; by conventional criteria and given the small sample size, this difference, however, is not statistically significant (two-tailed t-test; *p* =0.18). Further descriptive statistics of responders and non-responders can be found in Table 1.

**Table 1:** Descriptive statistics - responders and non-responders

	Responders (*n* = 20)		Non-responders (*n* = 33)	
	n (%)	MW (SD)	n (%)	MW (SD)
Gender				
male	13 (65%)		13 (39.4%)	
female	7 (35%)		20 (60.6%)	
Age		55.3 (13.08)		59.52 (11.32)
Years in school				
< 10 years	1 (5%)		1 (5%)	
10 years	10 (50%)		18 (56.3%)	
> 10 years	9 (45%)		13 (40.6%)	
not specified			1 (0.03%)	
Severity of lifetime depression^(a)^				
moderate	15 (75%)		14 (42.4%)	
severe	5 (25%)		19 (57.6%)	
PHQ-9		5.70 (3.84)		7.49 (5.03)
Use of professional help^(b)^				
yes	5 (25%)		9 (27.3%)	
no	15 (75%)		24 (72.7%)	

*Notes* (a) Severity of lifetime depression was assessed with the Munich-Composite International Diagnostic Interview (M-CIDI, [26]) in a face-to-face interview: occurrence of depression-relevant symptoms during a minimum period of two weeks within the previous life span was inquired. The severity of lifetime depression was classified as mild, moderate, or severe. Exclusion criteria were agreeing to questions about grief reactions and postpartum depression

(b) Utilization of professional help indicates previous use of medical or psychotherapeutic help and was assessed with the following item: “Have you ever received medical or psychological treatment due to (a) phase(s) of sadness/fatigue/indifference?” The variable was scaled dichotomously

As described above, we oversampled participants who stated that they did not seek professional help. In contrast to this prior intended imbalance of the sample regarding help-seeking, the analysis of the quantitative post-interview questionnaire later revealed deviations from the data collected by SHIP TREND-1: 5 of the initial 15 non-help-seeking participants had sought help by the time of the interview, resulting in a balanced sample (10 non-help-seeking and 10 help-seeking interviewees).

### Qualitative analyses

A summary of the key findings of our interview study can be found in Table 2 below. Following Table 2, we elaborate on our findings about coping with mental health problems and help-seeking. Moreover, we present respective relevant quotes and information about respondents.

**Table 2:** Key findings regarding barriers of help-seeking

	Findings	
1	Narrow concepts of mental illnesses and mental health services	Narrow concepts of mental illnesses and mental health services were frequent. The narrative of “lying on a couch” is still very common which is surprising considering the multitude of approaches in psychotherapy apart from psychoanalysis, to which the “couch”-narrative refers to. This “couch”-narrative seems to symbolize a lack of knowledge and experience, as well as outdated understandings with regard to mental health problems and professional treatment options such as psychotherapy.
2	Concealment, stigma and less pathologically perceived treatment offers	It was frequently discussed that mental health problems are often concealed from others which indicates that this is a common phenomenon in the investigated rural milieu. The idea of possibly being labelled as insane or crazy by others if one would seek professional help for mental health problems was a common expression of stigma. There seems to be a need for less pathologically perceived treatment offers such as “coaching” and “fairytale therapy” (i.e. stories that help to broaden perspective on problems and find solutions).
3	Traditional masculine norms inhibit help-seeking efforts	With regard to mental health, conformity to traditional masculine norms most likely inhibits help-seeking. Traditional masculinity beliefs point to structural and deeply rooted social conditions and a need for societal and individual transformation processes. This complex societal challenge, however, could only be addressed on multiple levels over a longer period of time.
4	Heterogeneity of motives to professional help-seeking	People with mental health problems have different motives for seeking professional help and differ in what outcomes they expect from treatment. Some interviewees primarily sought to improve symptoms and regain former function and performance – either through drug treatment, which acted as an accessible treatment option, or through more active participation in their own healing process. In contrast, others viewed therapy as an opportunity to mature and grow as well as to engage with the meaning of their lives and self-discovery.
5	Concerns about negative side-effects, e.g., deterioration of symptoms and loosing autonomy	Interviewees mentioned being concerned about deteriorating symptoms and losing autonomy due to psychotherapy. We consider these to be strong obstacles for seeking help.
6	Importance of family doctors in rural areas	Especially in rural areas, family doctors are important and often highly trusted first contact points for mental health problems. They also function as intermediaries who refer patients to psychiatric-psychotherapeutic treatment. Sometimes family doctors are perceived as a substitute for mental health professionals.

Following a data-driven analysis and grounded theory methodology, the following categories and aspects that influence help-seeking in a rural and structurally weak region emerged through the jointly conducted coding process. Some of these categories were found in several interviews. Others appeared only in few interviews but were so specific that they meaningfully contributed to the understanding of help-seeking.

#### Concepts of mental health, psychotherapy and mental health professionals

Interviewees showed different levels of knowledge, conceptions, and experiences regarding professional help for mental health problems. In general, we observed gaps in knowledge in the investigated rural milieu particularly regarding (1) reasons for the development of mental health problems, (2) existing treatment options for mental health problems, (3) how treatment for mental health problems works, and (4) what could be expected from treatment, such as possible benefits and/or side-effects.

Interviewees who had prior experiences – either directly or indirectly through their social network – expressed more positive attitudes towards mental health services. Some interviewees emphasized the professionalism and the importance of therapists after they have experienced treatment themselves.

And I know that they are professionals. So that - that’s not just at the beer table, but that’s really professionally done. Because I went through that myself and it helped me a lot. (Interviewee No.2, male, MHS user)^3^

In contrast, other interviewees did not seem to have a clear conception of psychotherapy or mental health professionals. For example, one of the interviewees used interchanging job titles for the same mental health professional throughout the entire interview (e.g., physiotherapist, psychotherapist, psychiatrist, doctor and physiologist).

Additionally, six interviewees described therapists and psychiatrists with colloquial wordings with negative connotations, such as “charlatan” and “Seelenklempner” (the literal English translation would be “soul plumber”). Psychotherapy was referred to as “[lying] on the typical couch”.

Well, I’d say, one knows it from the TV. You lie on the typical couch and the patient talks rather than the doctor giving concrete (…)^4^ – I don’t know, instructions or anything. But through his questions, he encourages people to think. (Interviewee No.11, male, no MHS^5^ use)^6^

#### Concealment of mental health problems

In terms of how interviewees cope with mental health problems or professional treatment, a central phenomenon that emerged was secrecy, i.e. concealing mental health problems from their mostly rural socio-environment. Secrecy seems to be quite common in the investigated rural milieu as indicated by frequent mention and discussion of the topic in the context of help-seeking.

Secrecy involves withdrawing from social contacts, avoiding an outward portrayal of one’s own health condition or of other’s health conditions, showing no weakness, and conforming to a desired image or social norm of being mentally healthy. Except for the partner and the immediate family, mental health problems are often concealed from others both by those affected and by close individuals.

I^7^: What were the reactions like, so did you tell anyone that you had sought treatment?

R^8^: Nope. And that has something to do with the uh, uh - with my last suicide attempt my wife was hiding, yes. (Interviewee No.5, male, MHS user)

One makes sure that no one from the outside notices it. I don’t know, it’s still in there like that [the term “in there” most likely refers to her pattern of thinking].^9^ Then, one is not necessarily in a position to talk about it when one is in it [“it” referring to the situation of sadness or helplessness]. (Interviewee No.10, female, MHS user)

Conversely, more openness towards disclosing mental health problems by peers can lead to a greater willingness to discuss one’s mental health problems as well.

[…] since I now know from two or three colleagues that they have similar problems, um, I can deal with it relatively openly. Um, if it wasn’t like that, that is, if I didn’t know whether someone also had such problems, I would probably keep it to myself, um, and rather want to get through it somehow. (Interviewee No.11, male, no MHS use).

#### Traditional masculinity norms

Particularly among middle-aged male interviewees in our sample, we observed that conformity to traditional masculine norms is a major phenomenon that influences help-seeking behavior and respective attitudes. This adherence to traditional masculine norms seems to be an important characteristic of middle-aged rural males and is reflected by (1) not discussing one’s health issues or “whining” about them and a perceived duty to endure hardships and suffering, (2) rationality, being solution oriented, and solving problems actively and alone, (3) a duty to work and (4) maintaining or regaining health-related functionality and performance. These aspects are elaborated in detail below.

##### (1) Toughness and the duty to endure suffering

Interviewees expressed not wanting to be perceived as whining or complaining and claimed that one simply has to accept symptoms and somatic treatments in life. An emphasis was placed on a general duty or requirement to accept and endure hardships and suffering, for example through bearing pain without complaint and not taking painkillers.

One talks about this and that, about the family, about the children and so on. And yes, you talk about that, but I don’t tell what - how I’m feeling right now - and whine, I don’t do that. I’m not the type who (…) - because my wife is also sensitive and I don’t want her to suffer when I feel such - such strong pain. (Interviewee No.13, male, no MHS use)

##### (2) Being a man is associated with rationality and proactively and independently finding solutions

 The majority of interviewed men emphasized not letting comfort, passivity (in the sense of “letting oneself go”), and an emotional “self-pity track” determine one’s actions, but rather proactivity, rationality, and goal- and solution-oriented behavior. These characteristics generally seemed to be attributed more to men than to women.

I try to approach things in a more solution-oriented way and (…) yes (…) let’s say, not to remain in this situation, i.e. to lament what - what - what is now, and rather to see what I can do now in this situation - what - what can I do now? (Interviewee No.4, male, no MHS use)

[The man is] the more deliberate one, compared to the other sex. He doesn’t operate quite so much based on his gut feeling. That is the typical difference, yes. (Interviewee No.20, male, MHS user)

Fittingly, a female interviewee mentioned that she generally perceives men as being more rational and less emotionally competent than women.

If it is things that somehow block me in my feminine ways of thinking, then I prefer a woman [i.e. female therapist]. Because I have the feeling that a man can’t understand this tangle of feelings and thoughts that a woman has. Because there are other plot lines, other - because women always connect a lot with emotions. It always has an emotional point somewhere, when women are thinking. I also notice that with my husband […]: the topic gets dealt with, done. […] (Interviewee No.12, female, no MHS use).

We noticed that especially male interviewees regarded disclosing mental health problems as whining or complaining, directly contrasting an active, self-reliant and solution-oriented attitude. They seemed to have an almost dichotomous understanding: one either solves problems alone or complains about them.

You can’t let yourself go. That’s my opinion, someone else might whine and complain, but that doesn’t get us anywhere (Interviewee No.13, male, no MHS use).

Help from others and professionals was often perceived as a last resort.

Let’s put it this way, I’m a loner… So, with the motto: “God helps those who help themselves”. Uh, the problems that I had in my life or, uh, in the family environment, I first try to sort by myself. (Interviewee No.6, male, no MHS use)

Suggestions of outdoor, physically active therapies like talking while walking or “physical exercise with conversation” were also mentioned in discussions about the desire to be active when tackling mental health problems. Another alternative treatment option mentioned only by male interviewees was “coaching” or a male “peer group” with similar problems.

I don’t see that necessarily in this - this pathological way now… so, one could say [I view it] more in the sense of coaching. “What thoughts do you have here?” and like “Come on, make something out of it now”. So, more clear assignments, right? So, and that might have been quite helpful. (Interviewee No.4, male, no MHS use)

From this point of view, coaching is seen as a less pathological approach for actively dealing with problems and avoids stigma attached to mental illness.

##### (3) Duty to work and its influence on help-seeking

 Interviewees discussed work as a coping strategy for problems and severe events in life, since being occupied with different assignments could function as a distraction or suppression of burdensome emotions and thoughts.

R: I had to accept it. There is nothing else I can do.

I: Is there anything that has helped you with it?

R: Work.

I: Work?

R: Mh. Yes. (…) Basically, suppressing the actual problem, right? (Interviewee No.6, male, no MHS use)

In our rural sample, work was identified as a frequent conversational topic and important identity-related concept for middle-aged male interviewees.

##### (4) Regaining health related functionality and performance

Among those who sought help, we observed different motives and expectations for treatment. Two male interviewees emphasized the use of professional help to regain functioning and performance in terms of health and participation. However, there were differences in terms of the orientation of their motivation.

##### Functioning through repair-medicine

One interviewee described his psychiatric treatment like repair medicine: mental health problems can be repaired with an external aid, such as antidepressant medication (Mirtazapine).

And I go there once a quarter and then we talk a bit, then I get my prescription and that’s it.

(Interviewee No.5, male, MHS user)

The prescribed medication seems to act as a quick, effective, and sufficient solution for his sleep problems.

[…] in the evening, I take my ‘kiss-my-ass tablet’ and then that works. Then I take my Mirtazapine and then later you notice when it slowly melts under your tongue and how you slowly fall into a dozy state. (Interviewee No.5, male, MHS user)

The effect that the medication has is described with partly figurative language, which suggests a more or less positive attitude towards the medication that helps to “function” in life, i.e. reaching tranquillity, ruminating less, and being more focused.

##### Performance orientation

In contrast to reaching basic functioning, another interviewee emphasized a health-related motivation. This interviewee focused on regaining a high-performance level with few to no symptoms so that he would be able to practice and enjoy physical activities such as wind surfing and mountain biking again.

I (…) draw my well-being in life from the fact that I can move, that I fly somewhere, drive somewhere, that I have my mountain bike […] that I am physically free. When it gets to the point that I can’t do a lot of things anymore, my – my will to live, I say […] then [my] life is clearly less worth living for me than for someone who [has] a family and who then sits in the chair […] (Interviewee No.20, male, MHS user).

#### Anticipated side effects of psychotherapy

##### Wound analogy: Professional psychotherapy could worsen a health condition

One interviewee mentioned a general tendency to try to look ahead instead of dwelling on things that happened in the past. Psychotherapy, in contrast, was described as a treatment that focuses mostly on confronting possibly unresolved issues from the past which could result in negative consequences such as unpleasant feelings and further exhaustion. Intense psychotherapy is seen as invasive and, like tearing open a wound, it would only worsen the experienced symptoms.

[With psychotherapeutic treatment] it’s like with – I think with any other medical treatment. It can lead to success, but it can also […] I think there’s a big unknown in it. Because also the – the preoccupation with the past, can certainly uh trigger different things in the person, right? And I think that is precisely the difficulty of psychotherapy. […] I think the field of psychotherapy is a much more difficult one [than the field of surgery] […] So it’s just as high a responsibility as if I give [a patient] an anesthetic, because […] the relationship – of course […] can trigger something that can really drive the person into - ultimately also into […] despair. (Interviewee No.4, male, no MHS use)

Following this, an alternative way of dealing with self-development and mental health problems was presented: engaging with problems of life through a more pleasant and less pathological approach through so-called fairy tale therapy (independent from professional help but with the help of books and together with friends).

Because [fairy tales] have, of course, also something quite sympathetic, or uh just [an] easy way to address these topics. And there is actually literature that, I say, combines psychology and fairy tales. (Interviewee No.4, male, use of MHS: no)

##### Difficulties with trust and an anticipated “boomerang” effect

Another anticipated barrier to help-seeking was being generally skeptical about new things and having difficulties to entrust personal problems and private thoughts to an unknown person, such as a psychotherapist.

There is a new person sitting in front of you and you have to reveal yourself somehow. And yes, that’s where the problems start again. That is with – I think one of the main problems is to be able to trust. […] (Interviewee No.10, female, MHS user).

In this context, it was also mentioned that after sharing troublesome or embarrassing thoughts with others, including friends, these information could later be used against oneself, even though this was considered to be an unreasonable fear. Overall, the fear of disclosing one’s mental health problems and trusting others seemed to be related to the fear of losing control and autonomy.

[…] if the thoughts might actually be rather unpleasant or perhaps embarrassing for me, then I don’t share them. Perhaps simply because of the certainly unfounded fear that it might come back as a boomerang and – in other words, in a way that you don’t really want it. (Interviewee No.10, female, MHS user)

##### Losing autonomy

Regarding seeking help for mental health problems, two interviewees (No. 4 & No. 10, see also the coding paradigms in the supplements B and C) expressed a strong need to maintain autonomy and the concern about being dependent on a therapist. Undergoing psychotherapy was perceived as almost equivalent to trading autonomy for dependence. In their understanding, help-seeking and dependence is connected to being helpless and weak, which does not fit their pride, habits, and self-concept. Therefore, they described to avoid seeking help for as long as possible.

[…] helplessness always has to do something with dependence. I would say, that I always tried to stay autonomous. [Later during the same interview:] So, [for me] that would be […] more the uh – kind of question or this – this “what’s happening?” or “what’s happening to me– “, giving up autonomy or something. (Interviewee No.4, male, no MHS use)

I: What do you think – you said the word “pride” several times now, where do you think does it come from?

R: I don’t know. Maybe you just don’t want to show that you are weak. Maybe it’s just misunderstood. (…) Because when you need help, that’s a sign: “I’m too weak, I can’t do it alone.” I always had to do everything alone. I think maybe it’s a matter of upbringing or a matter of getting used to.

I: Someone who needs help is weak?

R: Exactly (Interviewee No.10, female, MHS use).

For interviewee No.10, the negative association that seeking help for mental health problems has for her (i.e. needing help signifies weakness) only applies to herself. If others had mental health problems, she mentioned definitely wanting to help and strongly encouraging them to seek psychological or psychiatric help.

[…] he needs help, of course, and support and I would – I would also like to give that or I do give that if I am able to, but – through encouragement and whatever - but I don’t consider them weak. I only consider myself weak. That’s a problem but I think […] that affects a large part of our society. […] But yes, it only works in that direction. It doesn’t work in the other direction, at least not for me. It is difficult. (Interviewee No.10, female, MHS user)

Interviewee No.10 later added more information regarding her upbringing. The following quote illuminates how she perceives that her upbringing and parental care was connected to learned health behavior, such as dealing with health problems on her own:

R: With others you know immediately how you can help, whether you have to help, that you have to help and what all has to be done – only one’s own construction site… How do they say? “The cobbler has the worst shoes”, right?

I: (8 s) Now that you have just mentioned such a saying, um […] were there other guiding principles in your family related to health? […]

R: (Laughs) Yes, of course: “Don’t make such a fuss.” […] I’ve always been quite sickly actually […] I had pneumonia – I think I was a teenager at the time. […] But I had to get through that alone at the age of 14. There was just – the mother was not there; she worked or took care of the younger siblings. Therefore, one never really learned to care for oneself sensibly, only for others. (Interviewee No.10, female, MHS user)

From their perspective, psychotherapy is not only conceptualized as a dependent situation, but also involves different hierarchical levels in which the mental health professional is perceived as more powerful than the patient. Such a setting would not allow active patient participation, agency, or a trusting atmosphere and engagement at eye-level – all of which they described as being extremely important for them.

#### Importance of family doctors in rural areas

Apart from the regional vicinity and being socialized in the former GDR, interviewees who mentioned family doctors were fairly heterogeneous. Family doctors were considered (a) important first contact points for mental health problems, (b) intermediaries who refer patients to psychotherapeutic treatment, as well as (c) a substitute for mental health professionals in some cases.

- I: […] hypothetically speaking, in a case where you would need help, who would you turn to first?
  R: Well, my first contact person would be my family doctor, at least. One way or another. I have a good relationship with my family doctor. If I arrive there and say “Here, this and that is my ‘war condition’, what does the medical community have to say about it?”, then he would give me tips. (Interviewee No.15, male, no MHS use)
- I: How did you get into treatment back then? What was the first way to get there?
  R: Well, because of my family doctor. She practically gave me the – made the contact there. (Interviewee No.10, female, MHS user)
- With her [= family doctor], I was able to vent my anger a bit. So, she then more or less made the decision and also diagnosed for herself that it was not something really illness-related…physical illness, but rather a psychological thing and uh – yes. It was just that I knew that if something was wrong, I could definitely come back to her. So, I wasn’t branded [Later during the same interview:] So, I was really, really happy that I could go to my family doctor and that my family doctor was already so sensitive to consider a psychological approach […] I don’t know what it would have done to me if she had said: “Come on, you’re going to see a psychologist first! (Interviewee No.12, female, no MHS use)
  Yes, she once said in a very dry way that she – that we also have more psychological talks (laughs). So, it was rather – she took her time, right. Because then you could – it was already the talking with each other first, right?! So… and maybe I was lucky that talking [with her] was enough for me, right. (Interviewee No.14, female, no MHS use)

## Discussion

This qualitative study aimed to investigate conceptions of mental health problems, mental health services and important barriers to help-seeking with a focus on a rural milieu. Underlying motives, attitudes and values were subjects of interest during interviews with participants from the rural and structurally weak region of Western Pomerania. Apart from structural barriers such as low availability of professionals and long waiting times for an appointment for professional treatment, which are not the subject of our study, several attitudinal barriers and influences emerged: concepts of mental health problems, concerns about concealment and secrecy, masculinity norms, perceived side-effects of psychotherapy, and the role of family doctors.

### Mental health literacy, stigmatization and concealment of mental health problems

Generally, the interviewee group was quite heterogeneous regarding perceptions of mental health problems and professional help in their rural living environment. Attitudes towards mental health seemed to depend on whether or not interviewees had prior direct or indirect personal experiences with mental health services or mental health issues. Those who had prior experiences appeared to be more open-minded, positive, and aware about professional help and mental health issues. This corroborates findings of studies who also found that past treatment experiences are positively associated with help-seeking for mental health problems [e.g., [28–29]].

Other interviewees had lower mental health literacy [30–32] which was evident through pejorative mentions, little knowledge and indistinct, stereotypical or narrow conceptions about mental health problems, psychiatric-psychotherapeutic services, and mental health professionals. Poorer mental health literacy seems to be connected to older age and a more traditional upbringing where mental health received less attention than physical health resulting in fewer experiences with mental health problems in the past.

Our observations suggest that knowledge about when and how to address mental health problems is essential to be able to notice when to seek help. This is in line with previous work which showed that being able to recognize one’s mental health problems is an important prerequisite for seeking professional help [33]. This seems to be particularly challenging for older age due to poorer mental health literacy compared to younger adults [34, 35]. We suggest that inaccurate and outdated knowledge about psychiatric-psychotherapeutic services, especially among older people, should be addressed through contextually tailored mental health literacy campaigns and interventions.

We also noted that even though stereotypes were frequently prevalent, some interviewees sought help while others did not. Future research should investigate for whom stereotypes are likely to act as a barrier and under what conditions. Sometimes, interviewees identified the words they have used for describing mental health professionals as being outdated stereotypical narratives (“lying on the famous couch”) and put them into a broader context. Nevertheless, the words used first represent initial associations with. psychotherapy and professional help for mental health problems and can have a deeper meaning than just being synonyms or a stereotype. Using somewhat humorous words, such as “shrink” instead of “psychiatrist”, might be an attempt to downplay the perceived stigma of one’s own health condition.

Responses that implied negative attitudes towards professional help could be attributed to stigmatization: The anticipation of possibly being perceived as “crazy”, an “addict”, or a “nut job” if one were to seek professional help is likely associated with anticipated negative social consequences like discrimination and rejection or exclusion by others. For some interviewees, the anticipation of facing discriminating behavior was even stronger when they imagined being admitted to a psychiatric hospital.

Interviewees seemed to conceal their mental health problems in order to conform to their own self-concept and to perceived social norms. This might also be related to the extent to which individuals are generally able to recognize and express their own feelings. For this, easier routes to emotional disclosure could be helpful and encouraged in a variety of social environments (e.g., work places, health institutions, leisure activities and sports). Previous work has addressed this aspect through a carefully designed and evaluated intervention that addresses disclosing experiences with mental illness called “Honest, Open, Proud” [36–38].

Several respondents indicated that they consider psychotherapy to be useful and would encourage other people to seek help for mental health problems. However, when questioned about their own potential use of professional help for mental health problems, they appeared to consider professional help only as a last resort. This discrepancy between relatively progressive knowledge and favorable attitudes towards help-seeking and actual coping- and help-seeking behavior might be described as rhetoric modernization, a concept originally formulated by Wetterer in the context of modernization of gender issues [39]. Rhetoric modernization describes the phenomenon of how modernization of gender matters and gender equality takes place only rhetorically, i.e. even though new knowledge and attitudes have formed, they do not necessarily contribute to actual gender equality in practice.

Some interviewees seemed to regard psychotherapy as a dependent and hierarchical setting in which the patient follows the psychotherapist’s instructions somewhat passively and submissively. Such an understanding of psychotherapy poses an important barrier to using professional mental health services for affected individuals who particularly value autonomy.

### Traditional masculinity norms are barriers to mental health and well-being

Many aspects mentioned in the interviews of the study were consistent with findings of other studies looking at problematic help-seeking practices of men with mental health problems. For example, dominant masculinity norms discouraged disclosure of emotional distress and seeking help because it is considered ‘un-masculine behavior’ [e.g., 40] and men tend to downplay and trivialize mental health problems [41]. Findings of a meta-analysis and a systematic review have shown that conformity to masculine norms is negatively associated with both mental health and psychological help-seeking [42, 43]. In line with this research, we found that conformity to traditional masculine norms most likely acts as a barrier to help-seeking behavior by creating hesitation due to conflicting goals: wanting to maintain a masculine self-concept vs. wanting to end unpleasant symptoms. For female interviewees of our study this picture was more heterogeneous compared to men. We observed that gender norms appeared to be less influential for women regarding expression of emotions and mental health problems, as well as with respect to non-use of professional help.

#### Rationality, not complaining, and self-reliance are part of masculine self-concepts

Some narratives mentioned, mostly but not exclusively by male interviewees, indicate approval of traditional roles with binary notions of gender and dualistic Western ideas: Men allegedly being more rational and stronger compared to women, who were described as being more emotional and generally softer. Help-seeking is seen as passive and rather female, whereas finding one’s own solutions is seen as a proactive and male trait.

Traditional masculine norms are a characteristic feature of the studied milieu living in a structurally weak and less urban living environment. Narratives of enduring privations, maintaining and embodying strength in terms of physical and mental health as well as demonstrating toughness (e.g., referencing being a tough soldier), and being goal-oriented were frequently mentioned by middle-aged men. These narratives are important for middle-aged men and can be understood as a kind of self-affirmation of their identities in the context of traditional masculinity. Physical and/or mental health problems of middle-aged men are rarely disclosed to others in their social surroundings, possibly to fulfil or maintain the narrative of toughness and masculinity. As previously described by Latalova et al., depressed men who conform to masculine gender norms (e.g., by not crying) and think they should be able to cope with their mental health problems alone when they actually cannot, tend to stigmatize themselves which can inhibit help-seeking [44]. Maintaining toughness is a known dimension of masculinity and has been included as a dimension of “emotional control” in the Conformity to Masculine Norms Inventory (CMNI-22) which can be used to explore how a male client’s understanding of norms influences their relationships, work, and health [45, 46]. When others are not informed about potential mental health problems, the social environment cannot provide support. Social support, however, could act as a motivator for seeking and accepting professional help. The attitude of self-reliance fits western masculine norms and values (analogous to the dimension “self-reliance” in the CMNI-22) and is a known obstacle for seeking professional help for mental health problems [47]. Complementing to this, we observed that one reason for wanting to deal with problems alone is the belief that medical professionals have other, more important things to attend to than the interviewee’s “negligible” mental health problems. This unwillingness to consult medical personnel with only “minor” complaints has been documented in the past and seems to be connected with avoiding expression of weakness [48].

Internalized masculine gender norms, in the sense of not expressing emotions, seem to conflict with the use of mental health services where disclosure is often a necessary part. For men who conform to masculinity norms, taking antidepressant medication could serve as a middle ground between not wanting to disclose one’s mental health problems and not using mental health services at all as drug treatment allows for less active participation compared to psychotherapy. As proposed by some male interviewees, treatment options that are perceived as more physically active and less pathological might reduce perceived barriers to accessing mental health services. For middle aged and older men, coaching sessions, sport-based programs, or men-only peer-groups (see also [41, 49]) could be beneficial. Treatment offers might need to be better adapted to the lifestyle of rural men who tend to conform to masculine norms. Fittingly, Milner et al. have pointed to “the need for health literacy [media] campaigns that address the complexities of gendered help-seeking behaviors”, both for educating the target group and health professionals [47].

We would like to mention at this point, that professional help is not a universal remedy and might also not be suitable for everyone. Choosing not to seek professional help and to trust in self-healing or other forms of coping can also be a good choice for some with regard to quality of life [50] and should be acknowledged. For example, some cope with their mental health problems through sport and exercise [51], by talking to their partner, by distracting themselves with work or by applying “fairy tale therapy” to themselves as mentioned by interviewees in our study. Such coping strategies might be especially helpful in rural areas with a low density of psychiatric services.

#### Work often defines male identities. Men could benefit from a sensitive working environment

We observed that many male interviewees of the studied rural milieu perceive work as an important part of their life (analogous to dimension “primacy of work” of the CMNI-22). In the following, aspects of the work dimension are being described to further elaborate on characteristics of the male rural milieu.

Generally, working was mentioned as a coping strategy for mental health problems. This work-related coping strategy seems to partly substitute seeking professional help, as middle-aged and older men prefer to solve problems on their own. While this may be a suitable coping strategy for some, for others, this approach could worsen symptoms through exacerbating stress.

For middle-aged and older men, dedication to (hard) work was often perceived as a duty, with work being a necessary contribution to society and not just means to monetary enrichment and accumulation of material wealth. Work is an important ingredient for the functioning of a community. At the same time, work also has an integrative effect on an individual through enabling participation in a collective. This aspect is especially interesting in this context, as men in the interviews described seeing themselves as autonomous or even self-sufficient. Besides this societal and integrative function, work is also connected to individual benefits by establishing a certain status and image in society through social recognition of work performance, achievements, and certain professional positions. Accordingly, work functions as a central and defining part of men’s identities while also giving meaning, orientation, and purpose to life.

Having mental health problems and needing professional help contradicts traditional masculine norms. Since work is described as important to one’s functioning and identity, anticipated negative consequences for one’s standing at work might discourage men from seeking help or from openly admitting that they are having mental health problems. The importance that work has for middle aged men should be considered and used when aiming to improve help-seeking behavior in men. An open and appreciative working environment that allows men to express weaknesses and vulnerabilities seems of particular importance for prevention programs and further research.

#### Mental health services as means to regain functionality and performance

Motives for help-seeking and acceptance of mental health treatment among men included wanting to preserve or restore the ability to function and perform. In a qualitative study, O’Brien et al. expressed similar findings in which men embraced help-seeking more if it helped maintain or regain masculine activities [48].

We found that different levels of expectations towards mental health services and their outcomes exist. Some might wish for professional medical advice, simple solutions through medication, and the cessation of symptoms through treatment (i.e. medical repair service). Others, however, might prefer to work more actively with a mental health professional to find strategies to overcome mental health problems and regain the traditional masculine norms ideal of mental and physical fitness. Both approaches aim to reduce symptoms, allowing life to continue as before. This contrasts, for example, more existential motives that center around self-development and -exploration which, compared to the rural milieu, might be found more frequently in (younger) urban milieus.

### Anticipated side effects of psychotherapy can hinder help-seeking

Researching the risk of adverse events of psychotherapy is considered to be of integral importance [52] and receives increased attention in psychotherapy research [53]. For example, avoiding psychotherapy to stay independent demonstrates that psychotherapy is regarded as a setting in which one might become dependent on the therapist. In fact, therapy dependence is a negative effect that has been observed before [54]. Concerns about losing one’s autonomy during psychotherapy relates to the basic psychodynamic conflict between dependency and individuation. Concerns and needs of people with a strong desire to maintain their independence could be addressed early on, including the fear of being judged and the desire for communication at eye-level.

The analogy that psychotherapy may open a wound that has already healed relates to the known concept of deterioration or emergence of symptoms [54, 55]. We believe, the fear of adverse effects of psychotherapy should be taken seriously, with honest communication about possible benefits and negative side effects. At the same time, it is important to mention that negative side effects do not occur solely with psychotherapy, but also with other effective methods of treatment.

### Supporting family doctors in structurally weak rural areas

Our analyses of the interviews indicate that family doctors play a vital role in the process of seeking help for mental health problems in structurally weak and less densely populated rural areas such as Western Pomerania and are frequently perceived as an adequate replacement for mental health professionals. Interestingly, reports on family doctors by interviewees of our study were uniformly positive. This is in contrast with more inconsistent findings on the role of family doctors in a study in an urban setting where family doctors not only acted as a helpful gateway to mental health services but also as a deterrent to further help-seeking [41].

In rural areas, family doctors generally seem to function as a trusted first contact for mental health problems. In the context of rapidly changing technological and socio-political structures, such trusting and persisting relationships with health professionals appear to be particularly important, especially for people with conservative attitudes in a rural environment. Considering a socialization process that placed more importance on physical than on mental health, family doctors that also act as mental health professionals could help avoid or weaken stigma of mental illness. They might even be able to convince sceptical and traditionally minded patients to pursue further mental health treatment options. A similar suggestion on the supporting influence of health care professionals with regard to overcoming inhibiting traditional norms has also been made by Latalova et al. [44].

Against the background of anticipated stigma, family doctors can be seen as an easily accessible gateway for mental health services. This reasoning could be especially interesting for interventions in a rural setting. Due to fewer inhabitants it could be assumed that people in a village tend to know more about each other’s daily activities than people in large and more anonymous cities and it is not uncommon for a village to have only one family doctor to whom almost every villager goes to. Going to a family doctor cannot be directly associated with mental health problems since they also treat other health conditions.

Supporting family doctors caring for patients with symptoms of mental illness seems of particular importance in rural areas and we suggest further investigation regarding this matter.

### Limitations

This study is subject to various limitations which are outlined below:

- Qualitative data was analyzed and put into context through effort of a research team which was relatively homogeneous as three of the four German academics have a professional background in mental health. As much as diverging ideas were discussed to reach consensus in the team, interpretation of data remains biased as it is connected to similar privileges as well as structurally embedded and learned perspectives of the working group.
- Overall, there was a strong pre-selection of participants for this study. Of those who were invited, only about a third responded and participated in our interview study. Participants with severe depressive episodes in the past responded less frequently to the invitation, which could be due to possibly still existing depressive mood and the associated lower activity level.
- Due to high data protection requirements and regulations of the SHIP-study center, which managed access to the participants of the cohort study and invited all potential interviewees simultaneously, we were unable to follow theoretical sampling throughout the recruitment process. We tried to counteract this limitation regarding the flexibility of the sampling process according to Kruse [24] by deciding together as team which of the already transcribed interviews to analyze next based on initial analysis results.
- The study lacks generalizability because the participant group lives in a rural, structurally weak region and is quite old on average.
- It is possible that interviewees’ answers were, to some extent, influenced by social desirability, as awareness of the interviewers’ background in psychology may have influenced their perception about the relevance of professional help.

## Conclusions

Our findings suggest that barriers to help-seeking in rural areas are multifaceted. Help-seeking appears to be influenced by traditional masculinity norms, knowledge and beliefs about mental health problems and mental health professionals, as well as personal experiences.

The factors outlined in Table 2 are likely to influence help-seeking behavior in rural areas and could serve as helpful starting points for further in-depth research and intervention studies. Against the backdrop of our findings, we believe that (a) general improvements of mental health literacy, (b) treatment offers in settings that are perceived as less stigmatizing, and (c) tailored mental health care offers for men with strong traditionally masculine beliefs and mental health problems might help to improve mental health care in rural milieus. Alternative forms of coping with mental health problems through, e.g., exercise or disclosing one’s issues to a partner or peers should also be acknowledged.

### Electronic supplementary material

Below is the link to the electronic supplementary material.

**Supplementary Material (A–E)**. Overview of interview guideline (translated from German). Coding Paradigm (Example Interviewee No.4) – translated from German. Coding Paradigm (Example Interviewee No.10) – translated from German. Coding Tree (in German as interviews were conducted in native German language). Consolidated criteria for reporting qualitative studies (COREQ): 32-item checklist

## Data Availability

The data that support the findings of this study are available on reasonable request from the corresponding author, KV, and in consultation with the Department of Psychiatry and Psychotherapy, Medical Faculty, University Leipzig.

## Footnotes

1 German Democratic Republic.

2 Whether or not participants have sought professional help in the past was addressed by using a single and dichotomously (yes/no) scaled item (“Have you ever received medical or psychological treatment due to (a) phase(s) of sadness/fatigue/indifference?“).

3 The translation was kept as close as possible to the German wording.

4 Round brackets with three dots (…) indicate a three-second pause.

5 MHS…mental health services.

6 Interviewer comments such as „yes”, “okay”, “hm” were taken out of the quote for the purpose of reading flow.

7 I... interviewer

8 R... respondent

9 For a more comprehensible contextualization of some words or short statements of the interviewees, explanations in square brackets were occasionally inserted.
