# Supplementary material for: Value-related attitudes towards mental health problems and help-seeking barriers: a sequential mixed-methods design investigating participants with reported depressive episodes in rural Northern Germany with and without treatment experience
Source: BMC Psychiatry. 2024 Feb 22;24:153. doi: 10.1186/s12888-024-05521-9 (PMC10885433; doi:10.1186/s12888-024-05521-9)
Supplement: Supplementary file 1 — Supplementary Material (A–E). Overview of interview guideline (translated from German). Coding Paradigm (Example Interviewee No.4) – translated from German. Coding Paradigm (Example Interviewee No.10) – translated from German. Coding Tree (in German as interviews were conducted in native German language). Consolidated criteria for reporting qualitative studies (COREQ): 32-item checklist [file 12888_2024_5521_MOESM1_ESM.pdf]

Supplementary material A

Overview of interview guideline (translated from German)

I. Perception & assessment of health status + knowledge of mental health problems
II. Relationship with doctors & treatment experience
III. Dealing with problems & seeking support/opening up + psychiatric-psychotherapeutic treatment
IV. Social environment
V. Questions about overarching orientations/values for health and life in general

I. Perception & assessment of health status + knowledge of mental health problems

**Guiding questions / narrative prompt**		
(1) First of all, I am interested in what you consider to be health (Alternatively: What is a healthy life for you?)<br>(2) Please tell me what you are thinking of when you picture people who have mental health problems.<br>(Alternatively: What do you think distinguishes people with and without MHP?)		
**Checklist: content-related aspects**	**Questions for maintaining the conversation**	**Specific follow-up questions**
(1) Health status<br><br>• general well-being (descriptive)<br>• pain<br>• functional limitations<br>○ physical<br>○ mental/psychological	*(1) Health status*<br><br>non-verbal signals<br>backchanneling signals (hm, yes, okay)<br><br>○ What comes to mind? What do you think of?<br>○ Can you tell me a little more about that?	According to your statement on "health", how would you rate your current state of health? Alternative: How are you doing?<br><br>Apart from physical health, how are you doing mentally?
(2) Mental health problems<br><br>• subjective definition (own "image")<br>• if applicable, normative definition<br>• first symptoms (physical/psychological)<br>• importance of physical/psychological problems)<br>• experience (own/other persons)<br>• positive/negative statements	*(2) Mental health problems*<br><br>○ What else do you associate with mental health problems?<br>○ Is there anything else that comes to mind about mental health problems?<br>○ Can you tell me a little more about that?<br>○ Can you go into that a little more?<br>○ Do you maybe have an example of that so I can get a clearer picture?<br>○ Do you think of anything in particular?	What do you actually understand by mental health problems/ mental illnesses (What images come to your mind?)<br><br>How do you notice that something is physically/mentally a little off? ...if you think back, what were the first signs that you felt something was wrong?<br><br>If you imagine an old balancing beam, which would weigh heavier for you: physical or mental problems? Or would physical and mental problems weigh the same?

Note **Adapt choice of words to interviewee (psychological, mental, “spiritual/mental”)**

II. Relationship with doctors & treatment experience

**Guiding questions / narrative prompt**		
(1) What have been unusual [or difficult] experiences for you in connection with medical (/therapeutic) treatments?<br>(2) *Hypothetical question:* What would you wish from a doctor if you were in a really bad mental state?		
**Checklist: content-related aspects**	**Questions for maintaining the conversation**	**Specific follow-up questions**
(1) Unfamiliar/difficult experiences<br><br>• positive/negative experiences<br>• surprising<br>• consequences (private, professional, social environment, for future plans)<br>• consequences regarding health behavior: e.g. future utilization behavior	non-verbal signals<br>backchanneling signals (hm, yes, okay)<br><br>*(1) Unfamiliar/difficult experiences*<br>○ What else do you associate with difficult experiences in medical treatments?<br>○ Can you please give me an example of such an experience so that I can better imagine it?<br>○ Can you think of anything else?<br>○ Can you tell a little bit more about it?<br>○ Can you go into more detail about this?	What did you know in advance (of their treatment) about doctors/therapists involved in treating mental health problems?
(2) Wishes towards doctors in case of mental health problems<br><br>• relationship level	*(2) Wishes towards doctors in case of mental health problems*<br><br>○ What else would be important for you?<br>○ Is there anything else, …?<br>○ „You had mentioned earlier that… what do you mean by that?"	*...and in the case of mental health problems? How do you think good health care/treatment should look like best in your community?*

III. Dealing with problems & seeking support/opening up + psychiatric-psychotherapeutic treatment

<table>
<tr><td colspan="3">Guiding questions / narrative prompt</td></tr>
<tr><td colspan="3">(1) Thinking back to the last situation you can remember in which you felt very sad/helpless, what was that like for you? What did you do then?
(2) What comes to your mind when you think of psychotherapy and/or psychiatric treatment?</td></tr>
<tr><td>Checklist: content-related aspects</td><td>Questions for maintaining the conversation</td><td>Specific follow-up questions</td></tr>
<tr><td>(1) Dealing with mental health problems
• coping strategies
• talking about it with other people
→ friends, family, …
→ Who to confide in?
→ What is talked about? (emotions/ thoughts/ sorrows/ pain…)
• Professional support (family doctors/ psychotherapist/psychiatrist)
• general attitude towards help-seeking</td><td>non-verbal + backchanneling signals

(1) Dealing with mental health problems

○ And apart from that? Is there anything else they do when you're not feeling well mentally?
○ Can you tell me a bit more about that?
○ Could you go into a little more detail about that, please?
○ Do you have an example for that?</td><td>Do you talk to other people about what is bothering you? Do you share what's going on inside you?
- Can you give an example here: What do you talk about then? → feelings, thoughts?

Would you seek professional help if you were having a really bad time emotionally?

In general, how easy or difficult is it for you to ask for help in different areas of your life?</td></tr>
<tr><td>(2) Mindset regarding treatment for mental health problems

• subjective definition
• if applicable, normative definition
• treatment experiences (own / of others)
• readiness for psychotherapy
• expectations towards practitioners
• psychotherapy meaningful / useful / effective?
• drug / non-drug treatment options?
• positive / negative statements regarding professional treatment</td><td>(2) Mindset regarding treatment for mental health problems

○ Can you think of anything else about psychotherapy or psychiatry?
○ Is there anything else that you associate with it?
○ ...can you elaborate a little bit further, please?
○ Is there anything else that you associate with the treatment of mental health problems?</td><td>• What would you advise someone who has mental health problems to do?

• What do you think happens during psychotherapy?
• How useful do you think psychotherapy is?
• What do you have in mind when you think of people going through psychotherapy?
• Have you been in psychotherapeutic treatment yourself → How was/is that for you?
• Can you imagine doing psychotherapy yourself if you were mentally unwell?
- <u>yes:</u> What would be good for you? What should the therapist do? Desires? Where would you feel more comfortable (w/m)?
- <u>no:</u> What would hinder you?

• What do you think about psychopharmaceuticals (e.g. antidepressants)?</td></tr>
</table>

IV. Social environment

**Guiding questions / narrativ prompt**		
(1) What are your experience with mental health problems in your circle of acquaintances? ...has anyone ever been at or beyond their mental health limits?<br>(2) How do you understand the phrase "being a real man"/"being a real woman"?		
**Checklist: content-related aspects**	**Questions for maintaining the conversation**	**Specific follow-up questions**
(1) Friends<br>• Dealing with mental health problems<br>• openness<br>• support<br>• attitudes	non-verbal signals<br>backchanneling signals	• What do you think your friends/ family/ colleagues think about mental health problems?<br>• What do you think your friends would say to you if you were on sick leave (due to a mental illness) for several months?<br>• (What would it be like for you to ask your friends and family for support if you were in a really bad mental health situation?)
(2) Gender roles<br>• Are there typical male / typical female characteristics?<br>• roles? tasks?<br>• similarities? differences?<br>• financial aspects / financial autonomy?<br>• importance of work<br>• competitive thinking<br>• showing weakness<br>• overload → How to deal with it?	*<u>Choice of third gender option ← ← ← ← ← ←</u><br>“Since the end of 2018, intersexual people in Germany have the possibility to choose the option "diverse" in addition to the genders "male" and "female" when entering the civil status register. What do you think about that?”	• How important is your work in your life/success in your work to you?<br>• How important is it to you to be better than others at what you do?<br>• How important is it for you to be able to financially provide for yourself independently of others?<br>• Is there anything that’s typical male or typical female?<br>• What is your opinion of the third gender option?*<br>• Do you know the feeling of being overloaded? How do you deal with it?<br>• Can you tell me how you deal with your feelings?<br>• How do you feel about people showing weakness?

IV. Questions about overarching orientations/values for health and life in general

- When you think about your life so far and about your current life, are there certain principles that you try to live by or that guide your actions every now and then?
- When it comes to dealing with illness are there any guiding principles that have played a special role in your life?
- From your own life experience, what advice would you give to young people for life/for their health?
- If you have children (or would have children), are there certain pieces of wisdom or values of which you are convinced and which you would like to pass on to your offspring?
- Are there certain qualities that your parents or other relatives have that you value and would like to preserve?

Before ending the interview:

Is there anything we forgot to talk about during the interview?
…something that has been missed out so far, but is important for you to mention?

Supplementary material B

Coding Paradigm (Example Interviewee No.4) – translated from German

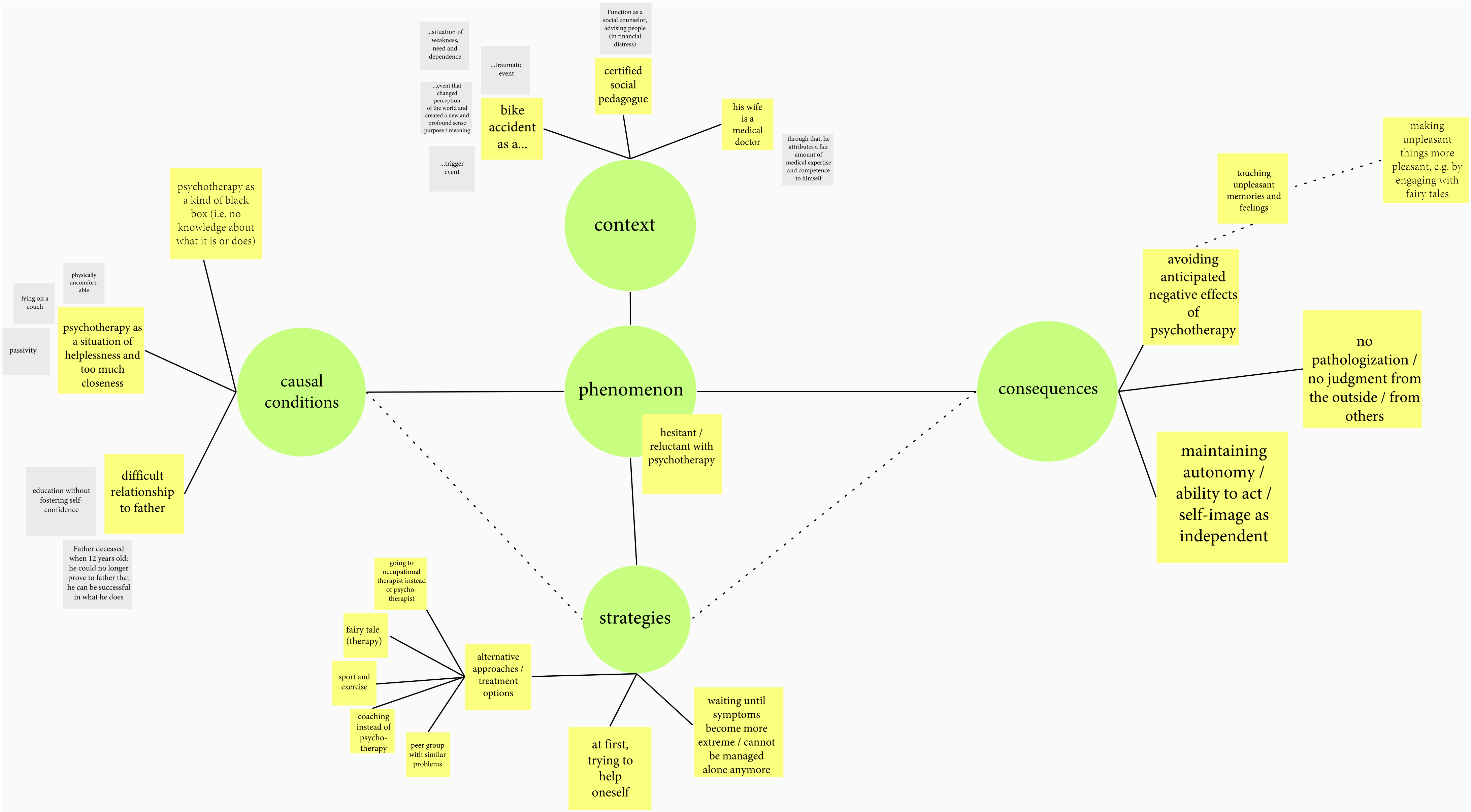

Supplementary material C

Coding Paradigm (Example Interviewee No.10) – translated from German

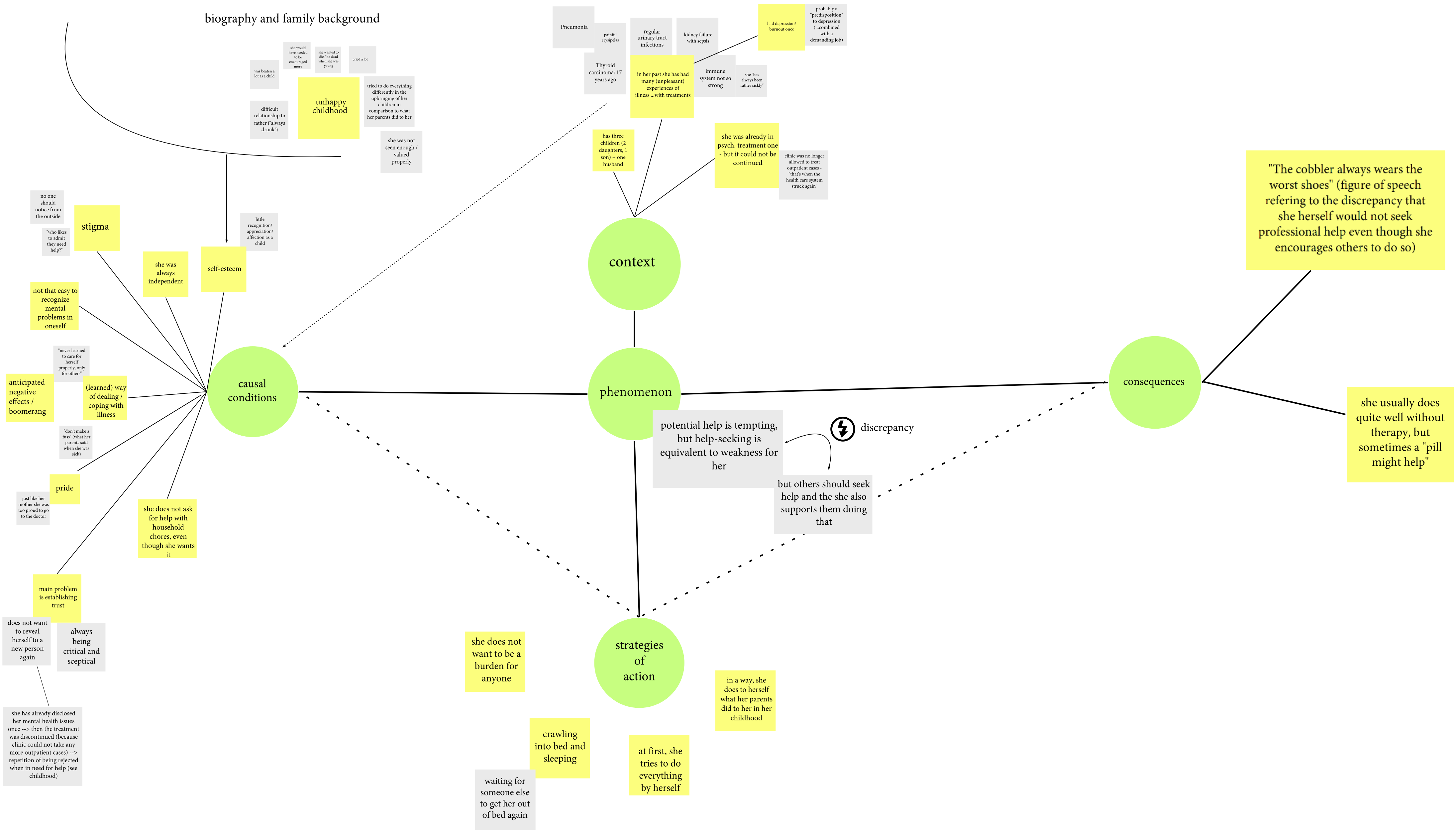

Supplementary material D

Coding Tree (in German as interviews were conducted in native German language)

- Gesundheit & gesundheitsbezogene Einstellungen
  - Verständnis von Gesundheit
  - Ressourcen/Schutzfaktoren
  - Risikofaktoren
  - Körperliches
    - Erkrankungen/Einschränkungen
    - Sorgen
    - Eigene körperliche Grenzsituationen
  - Psychisches
    - Eigene psychische Probleme
    - Umgang mit psychischen Problemen
    - Sorgen
    - Verständnis/Bilder von psychischen Problemen/Erkrankungen
    - Außenkommunikation von psychischen Problemen
    - Ursachenvorstellung
    - Psychische Probleme anderer / Erfahrungen
  - Einschätzung eigener Gesundheit
  - Behandlung / Assoziationen zu Behandlungsansätzen
- Hilfe suchen/annehmen
  - Unterscheidung zwischen selbst und anderen
  - im Alltag / allgemein
  - professionelle/psych. Behandlung (Inanspruchnahme)
    - Zweifel / Hindernisse
    - etwas ist komplett falsch / ich komme hier nicht mehr weiter
    - keine Kenntnisse vor Behandlung
    - erster Kontakt mit Hilfsangeboten
    - legitim und nötig, Hilfe zu suchen, wenn man nicht weiterkommt
    - (psych.) Hilfe in Anspruch nehmen ≠ Schwäche
    - Interesse an Psychologie
    - dankbar
    - hilfreich/sinnvoll
    - professionell
- Werte / Haltungen / Orientierung
  - allgemein
    - Offenheit / Wertfreiheit / Vorurteilsfrei
    - Gemeinsamkeit
    - Potenziale ausschöpfen (+)
    - Harmonie / liebend / freundlich
    - Unterhaltung als Gemeinschaftswert
    - Tapferkeit
    - Disziplin/Kraft&Stärke/Leistung/pflichtbewusst
    - Gerechtigkeitsverständnis
    - Individualismus (jeder ist anders/individuell)
    - Finanzielles / Materielles
    - Positive Sicht auf Krisen (Krise als Chance)
    - politisches Interesse
    - Empathie
    - Respekt/Achtung erfahren
    - Einfluss ausüben
    - Verantwortungsbewusstsein
    - Ehrlichkeit zu sich selbst (einsichtig sein)
    - Meinungsfreiheit
    - Autonomie/Eigenständigkeit
    - Hilfsbereitschaft
    - kritisch sein / hinterfragen
  - Geschlechterkategorie / Gender / Traditionelle Rollen / divers
    - unsicher / indifferent
    - Rollen-/Aufgabenverteilung
    - über Frauen
    - über Männer
    - Aspekte der Selbstreflexion
  - Arbeit
- Emotionalität & psychischen Belastungen
  - Eigenes Verständnis/Zugang zu eigenen Emotionen
  - Umgang mit Emotionen/psychischen Belastungen
  - Kommunikation von (mentalen) Problemen
  - neg. Selbstbild
  - Fremdwahrnehmung (wie empfinden andere Personen Emotionalität)
- Biografie
  - Höhen und Tiefen im Leben
  - Externe Bedingungen
  - Familiäre Vergangenheit
  - Kindheit
  - Prägende Erlebnisse / Lebenswandel / Umdenken
  - Studium/Ausbildung
- Sozialgefüge
  - Familie / Familiäres Umfeld
  - Erweitertes soziales Umfeld

Consolidated criteria for reporting qualitative studies (COREQ): 32-item checklist

Consolidated criteria for reporting qualitative studies (COREQ): 32-item checklist

Please indicate in which page each item has been reported in your manuscript. If you do not feel an item applies to your manuscript, please enter N/A.

For further information about the COREQ guidelines, please see Tong *et al.*, 2017: https://doi.org/10.1093/intqhc/mzm042

**No.**	**Item**	**Description**	**Reported on Page No.**
**Domain 1: Research team and reflexivity**			
Personal characteristics			
1.	Interviewer/facilitator	Which author/s conducted the interview or focus group?	4
2.	Credentials	What were the researcher's credentials? *E.g. PhD, MD*	3
3.	Occupation	What was their occupation at the time of the study?	3
4.	Gender	Was the researcher male or female?	4
5.	Experience and training	What experience or training did the researcher have?	4
Relationship with participants			
6.	Relationship established	Was a relationship established prior to study commencement?	No
7.	Participant knowledge of the interviewer	What did the participants know about the researcher? *E.g. Personal goals, reasons for doing the research*	4
8.	Interviewer characteristics	What characteristics were reported about the interviewer/facilitator? *E.g. Bias, assumptions, reasons and interests in the research topic*	7,14
**Domain 2: Study design**			
Theoretical framework			
9.	Methodological orientation and theory	What methodological orientation was stated to underpin the study? *E.g. grounded theory, discourse analysis, ethnography, phenomenology, content analysis*	2-5
Participant selection			
10.	Sampling	How were participants selected? *E.g. purposive, convenience, consecutive, snowball*	3
11.	Method of approach	How were participants approached? *E.g. face-to-face, telephone, mail, email*	3-4
12.	Sample size	How many participants were in the study?	5-6
13.	Non-participation	How many people refused to participate or dropped out? What were the reasons for this?	5-6
Setting			
14.	Setting of data collection	Where was the data collected? *E.g. home, clinic, workplace*	4
15.	Presence of non-participants	Was anyone else present besides the participants and researchers?	4

16.	Description of sample	What are the important characteristics of the sample? *E.g. demographic data, date*	5-6
Data collection			
17.	Interview guide	Were questions, prompts, guides provided by the authors? Was it pilot tested?	3-4
18.	Repeat interviews	Were repeat interviews carried out? If yes, how many?	No
19.	Audio/visual recording	Did the research use audio or visual recording to collect the data?	4
20.	Field notes	Were field notes made during and/or after the interview or focus group?	4
21.	Duration	What was the duration of the interviews or focus group?	4
22.	Data saturation	Was data saturation discussed?	5
23.	Transcripts returned	Were transcripts returned to participants for comment and/or correction?	No
**Domain 3: analysis and findings**			
Data analysis			
24.	Number of data coders	How many data coders coded the data?	5
25.	Description of the coding tree	Did authors provide a description of the coding tree?	yes (supplement)
26.	Derivation of themes	Were themes identified in advance or derived from the data?	4-5
27.	Software	What software, if applicable, was used to manage the data?	5
28.	Participant checking	Did participants provide feedback on the findings?	No
Reporting			
29.	Quotations presented	Were participant quotations presented to illustrate the themes / findings? Was each quotation identified? *E.g. Participant number*	6-11
30.	Data and findings consistent	Was there consistency between the data presented and the findings?	6-11
31.	Clarity of major themes	Were major themes clearly presented in the findings?	7
32.	Clarity of minor themes	Is there a description of diverse cases or discussion of minor themes?	7-10

Developed from: Allison Tong, Peter Sainsbury, Jonathan Craig, Consolidated criteria for reporting qualitative research (COREQ): a 32-item checklist for interviews and focus groups, International Journal for Quality in Health Care, Volume 19, Issue 6, December 2007, Pages 349–357, https://doi.org/10.1093/intqhc/mzm042
